# Infectious Diseases among Refugee Children

**DOI:** 10.3390/children6120129

**Published:** 2019-11-27

**Authors:** Avinash K. Shetty

**Affiliations:** Department of Pediatrics and Office of Global Health, Wake Forest School of Medicine and Brenner Children’s Hospital, Medical Center Blvd, Winston-Salem, NC 27157, USA; ashetty@wakehealth.edu; Tel.: +316-713-4500; Fax: +316-716-9699

**Keywords:** refugee, migrant, children, infectious diseases, screening, immunizations

## Abstract

In recent years, there has been a substantial increase in refugee and asylum-seeking adults, adolescents and children to high-income countries. Infectious diseases remain the most frequently identified medical diagnosis among U.S.-bound refugee children. Medical screening and immunization are key strategies to reduce the risk of infectious diseases in refugee, internationally adopted, and immigrant children. Notable infectious diseases affecting refugee and other newly arriving migrants include latent or active tuberculosis, human immunodeficiency virus type 1 (HIV), hepatitis B, hepatitis C, vaccine-preventable diseases, malaria, and other parasitic infections. The U.S. Centers for Disease Control and Prevention and the American Academy of Pediatrics have published guidelines for health assessment of newly arriving immigrant, refugee, and internationally adopted children. Although, data on the health risks and needs of refugee exists in some high-income countries, there is an urgent need to develop robust evidence-informed guidance on screening for infectious diseases and vaccination strategies on a broader scale to inform national policies. Innovative approaches to reach migrant communities in the host nations, address health and other complex barriers to improve access to high-quality integrated health services, and strong advocacy to mobilize resources to improve health, safety, and wellbeing for refugee children and their families are urgent priorities.

## 1. Introduction

In recent years, there has been a substantial increase in refugee and asylum-seeking adults, adolescents, and children to the European Union (EU)/European Economic Area (EEA) and the U.S. [1,2,3,4,5]. The Global Trends Report of the United Nations High Commissioner for Refugees (UNHCR) reported that by the end of 2017 approximately 68.5 million people were forcibly displaced across the globe, including 25.4 million refugees, 40 million internally displaced persons (IDPs), and 3.1 million asylum seekers [6]. By United Nations Children’s Fund (UNICEF) estimates, 50 million children migrated across borders or suffered forced displacement within their own nations in 2015 [7]. More than 28 million children had to flee from their homes because of wars, violence, and insecurity [2]. Between 2010 and 2015, there has been a dramatic increase (75%) in the number of child refugees, with important implications for health care services [7].

Across Europe, children account for more than 30% of all asylum-seeking people [5]. In EU/EEA countries, an estimated 200,000 to 400,000 children sought asylum annually from 2015 to 2017; an additional 800,000 asylum-seeking children and adolescents arrived in 2015 to 2016 [1]. Asylum-seeking and refugee children from Syria, Afghanistan, and Iraq were the single largest group resettled in the EU/EEA in 2016 and 2017 [1,6]. Germany was the top country for asylum-seeking and refugee children in the EU and EEA followed by France, Greece, Italy, Austria, Sweden, the United Kingdom, Spain, and Switzerland [1]. 

The United States Citizenship and Immigration Services (USCIS), an agency nested under the Department of Homeland Security defines a *refugee* as an individual outside of his or her country of a nationality who is unable or unwilling to return to their native country because of persecution or a well-founded fear of persecution. An *asylee* is defined as an alien in the U.S. or at a portal of entry who is found to be unable or unwilling to return to native country because of persecution or a well-founded fear of persecution [3,8,9,10,11]. Refugee and asylum seekers are granted legal status in the U.S. Refugees typically undergo screening for resettlement outside of the U.S. whereas asylum seekers are physically in the U.S. at the time of their application submission [12]. Immigrant children are defined as children who are foreign-born, or children born in the U.S. who reside in a household with at least onee parent who is foreign-born [10].

In the U.S., from 2004 onwards, the number of refugee admissions per year has varied over the years, ranging from 41,094 to 74,602 annually, with 69,909 arrivals in 2013 [13]. In 2016, the number of refugee arrivals peaked at 84,994 followed by 53,716 admissions in 2017 [13]. Data from the U.S. State Department’s Worldwide Refugee Admissions Processing System (WRAPS) indicates that the number of refugee admissions declined significantly to 22,491 in 2018 (a 58% decrease from 2017) [13] (Table 1).

During 1 October 2018 through to 30 April 2019, approximately 15,000 refugees were re-settled in the U.S.; of these, women and children aged less than 14 years represented 58% (8521) of refugee arrivals from the top five countries (Democratic Republic of Congo, Burma, Ukraine, Eritrea, and Afghanistan) [13]. In the first half of 2019, the top refugee-receiving states in the U.S. were Texas (9%, or 1389 individuals) and New York (6%, 932) followed by California (6%, 848) and Washington (6%, 827); other states including North Carolina (662), Ohio (651), Kentucky (649), Georgia (589), Michigan (584), and Arizona (536), accounted for 4% each. Overall, these 10 states represented 52% of all refugees resettled during the first half of 2019 [13]. Considerable variation exists among states by refugees’ country of origin. For example, Burmese refugees historically were the single largest group resettled in the U.S. between 2007 through to April 2019 but were in the top group in only 19 U.S. states [13]. In contrast, 12 states, including California and Michigan, admitted more Iraqi refugees than any other country of origin during the past decade whereas Florida and Nevada have resettled more Cuba refugees than any other nationality group [13].

Newly arriving refugees and asylum seekers often arrive from low-income countries plagued by war and social conflicts, natural disasters, and economic challenges, and experience long journeys [14]. Poor health systems in the setting of conflicts can result in low vaccination coverage for children; in addition, history of vaccination and documentation of prior vaccine receipt is often incomplete [15,16]. In one cohort study of 2126 asylum-seeking children to Denmark, 30% were unimmunized based on the Danish immunization schedule [17]. In addition, the risk of communicable diseases is high given overcrowded circumstances during long migration journeys to Europe and other countries [18]. Refugees also face a myriad of challenges, including high mobility, poor living conditions, and barriers to access quality health care [10,19,20,21]. In addition, lack of interpretation services, cultural and language differences, limited health literacy and knowledge about health, lack of awareness regarding human rights, unfamiliar health systems, and lack of preparedness of health care providers in high-income countries to address the health and complex social issues of refugee populations further compound the problem [10,19,21].

Infectious diseases remain the most frequently identified medical diagnosis among refugee children arriving in the U.S. [22,23]. In addition, other key priority health conditions affecting refugee children is summarized in Table 2. Healthcare providers caring for refugee children must be aware of communicable diseases that are endemic to the refugee’s country of origin. Priority infectious diseases affecting refugees and other newly arriving migrants to high-income countries include tuberculosis (TB) (active and latent), HIV, hepatitis B, hepatitis C, vaccine-preventable diseases (such as measles, mumps, rubella, diphtheria, tetanus, pertussis, and *Haemophilus influenzae* type b), and parasitic infections (such as strongyloidiasis and schistosomiasis) [4,22,23,24]. In the EU and EEA, infectious diseases are the most common cause of illness in migrant children living in refugee camps and other reception areas, including acute respiratory tract infections, outbreaks of vaccine-preventable diseases, such as measles, and skin infection (e.g., scabies, pediculosis); gastrointestinal infection (e.g., shigellosis); typhoid fever; hepatitis A; tuberculosis; and malaria [25,26].

In this article, we discuss the priority infectious disease affecting refugee children, review guidelines for evaluation and screening tests, and address preventative measures through immunizations.

## 2. Evaluation and Screening for Priority Infectious Diseases

Medical screening and immunization are key strategies to reduce the risk of infectious diseases in refugee, internationally adopted, and immigrant children [23,27,28,29]. The U.S. Immigration and Nationality Act (INA) mandates a medical screening examination performed by a designated civil surgeon and panel physicians for all refugees to identify inadmissible health conditions (e.g., imported communicable diseases). Inadmissible infectious diseases include: A) Communicable diseases of public health significance: Active tuberculosis (TB), syphilis (infectious stage), gonorrhea, and leprosy (infectious stage); B) communicable diseases that are listed as quarantine diseases in a presidential executive order: Cholera, diphtheria, plague, smallpox, yellow fever, viral hemorrhagic fevers, severe acute respiratory syndrome (SARS), and influenza caused by novel or re-emergent influenza (pandemic flu); and C) other infectious diseases designated as public health emergencies of international concern to the World Health Organization (WHO): Polio, smallpox, severe acute respiratory syndrome (SARS), influenza, and Ebola [12,29].

Compared to internationally adopted children, refugee children often undergo general medical screening (including a physical examination) in an organized fashion before the issue of an emigration visa pre-arrival in the U.S. [27,28,29,30,31,32]. Screening tests performed for U.S.-bound refugee children include serologic testing for syphilis, and tuberculin skin test or interferon gamma-release assay (child ages 2–14 years), and chest X-ray (for all applicants aged 15 years and older). In this setting, screening tests are often reliable since the screening process is coordinated by established reputable organizations [27]. In addition, access to primary prevention strategies, including immunizations, vitamin supplementation, and dental care, is generally more consistent with refugee children compared to internationally adopted children [27]. Certain tropical infections (e.g., malaria, filariasis, typhoid fever, schistosomiasis) are more frequently encountered in refugee children compared to internationally adopted children from countries outside sub-Saharan Africa [27].

The examination site and location is overseas for U.S.-bound immigrants and refugees and performed by panel physicians. A panel physician is a physician located outside the U.S, and authorized to conduct pre-immigration medical screening (for those individuals who apply for immigrant or refugee status prior to arrival in the U.S.). The U.S. Department of State selects the panel physicians. The U.S. Centers for Disease Control and Prevention (CDC)’s Division of Global Migration and Quarantine (DGMQ) provides technical instructions to civil surgeons and panel physicians who perform the mandated medical examinations for migrants [29]. The medical examination consists of a history and physical examination; laboratory screening tests for Tuberculosis (TB), syphilis, and gonorrhea; assessment of immunization status; diagnosis of mental health problems posing a danger to self or others; screening for substance abuse; and vaccinations for immigrants [33]. In addition, the CDC also provides pre-departure medical screening guidelines and health interventions for refugees based on risk in the country of origin, fitness to fly, and availability of resources and logistical support [29,34].

The U.S. CDC has published guidelines for the U.S. domestic medical examination for newly arriving refugees. [31]. Federal regulations do not require repeat medical screening examination for refugees upon arrival in the U.S. However, the Department of Health and Human Services recommends that all refugees have a post-arrival comprehensive assessment of health status and review of vaccinations by any qualified health care provider. The domestic medical assessment usually occurs 1 to 3 months after arrival as a coordinated effort between resettlement volunteer agencies and state public health departments [29]. Physician(s) at health departments who fulfill the legal requirements of a civil surgeon may participate in physical examination and vaccination assessment of refugees. A civil surgeon is a licensed U.S. physician with more than 4 years of experience and authorized by U.S. Citizenship and immigration Services (USCIS) to perform medical examinations [35]. CDC’s post-arrival guidelines for refugee screening are available at [31]. Health care providers are encouraged to review site-specific clinical protocols via their local or state health department [29]. In addition, health profiles of diverse U.S.-bound refugee populations are maintained and updated by the CDC/Division of Global Migration and Quarantine (DGMQ) to inform health care providers regarding common health issues of newly arriving refugee groups [29].

The American Academy of Pediatrics recommends medical screening for all newly arrived refugee children and linkage to primary care as soon as possible after arrival [28]. The American Academy of Pediatrics (AAP) immigrant child health toolkit is a valuable resource for pediatricians caring for immigrant, refugee, and internationally adopted children [10,11,36]. The components of the AAP immigrant child health toolkit comprises of: Key facts, clinical care (e.g., medical screening, treatment recommendations for newly arrived immigrant children), mental and emotional health, access to health care and public benefits, immigration status and related concerns, state legal resources for immigrant children and families, and advocacy [10,36]. Screening tests for evaluation of common infectious diseases in refugee children are depicted in Table 3 [23,29]. The resources for clinicians caring for refugee children are summarized in Table 4.

### 2.1. Human Immunodeficiency Virus Type 1 (HIV)

HIV remains a major global public health challenge in many countries and disproportionately affects key vulnerable populations such as young men, women and children. Sub Saharan Africa bears the brunt of the HIV pandemic. 

In 2018, 37.9 million (32.7 million–44.0 million) people worldwide were living with HIV including 1.7 million (1.3 million–2.2 million) children (<15 years) [37]. An estimated 20.6 million (18.2 million–23.2 million) people living with HIV reside in eastern and southern Africa [37]. From 1999 through to 2006, migrants, predominantly from sub-Saharan Africa, accounted for over half of people living with HIV in the 27 European Union countries [38].

The prevalence of HIV infection in newly arrived migrant children depends on the risk factors from their countries of origin (such as prevalence of maternal HIV infection and risk of mother-to-infant HIV transmission, maternal drug use, receipt of blood products) [5,23]. Studies from Germany, Italy, and Canada have documented an HIV prevalence of 0.4% to 2% among migrant children [39,40,41].

From 2010, routine HIV testing as part of the medical evaluation for immigration is not required for refugees and immigrants prior to arrival in the U.S. [28]. However, HIV testing is recommended for individuals who are diagnosed with TB disease as part of their medical outside of the U.S. After arrival in the U.S. the CDC recommends HIV testing for refugees aged 13 through to 64 years; HIV testing is encouraged for children aged younger than 13 years and adults older than 64 years [42]. Many factors determine the need to routinely screen for HIV infection in newly arrived migrant children in the U.S., including history, risk factors, physical examination findings, and HIV prevalence in the country of origin [23]. Some experts suggest that HIV testing may be indicated for most immigrant children [43].

Refugee children of any age with clinical suspicion for HIV should undergo testing. In infants and children younger than 18 months of age, virologic tests (HIV DNA or RNA assays) are recommended to screen for HIV infection since HIV antibody tests are unreliable due to the persistence of transplacental acquired maternal antibodies [23]. In contrast, in children aged 18 months and older, the diagnosis of HIV infection can be made by serology. If the diagnosis of HIV infection is confirmed by two-tier testing, referral to a pediatric infectious disease specialist for appropriate treatment and further evaluation is recommended [23].

### 2.2. Tuberculosis

Migrants often arrive from countries with a high incidence of TB [5,44]. In addition, the long travel and housing in overcrowded settings during re-settlement increases the risk of exposure to TB [45]. Classic symptoms of TB disease in children include fever, weight loss, and chronic, non-remitting cough. The most common forms of TB disease in children are pulmonary disease (hilar and mediastinal adenopathy, parenchymal disease) followed by extrapulmonary disease (tuberculous lymphadenopathy, meningitis, military disease). Compared to adults, extrapulmonary TB is more common in children [46]. Infants and young children are at higher risk of TB disease and progression to severe forms (such as tuberculous meningitis and military TB) following infection (latent disease) [46]. Therefore, screening for latent TB infection and early diagnosis and treatment of TB disease is crucial to reduce morbidity and mortality [47]. Studies from EU/EEA have reported a higher incidence of active TB is higher in migrant children compared with non-migrant children [5,48,49]. In one study from Germany, of 968 asylum-seeking children screened for TB, 66 (6.8%) children were diagnosed with TB infection (58 latent TB infection, 8 active TB) [48]. Studies report that approximately 1% of migrant children have evidence of active TB; rates up to 8% have been reported among newly arrived refugee children in the U.S. [50,51,52,53,54].

The U.S. revised pre-departure TB screening guidelines for immigrants in 2007 followed by complete implementation on a country-by-country basis in 2013 [29] (Table 3). Screening tests for TB are determined by the age of the child and HIV status and include the tuberculin skin test (TST) and interferon gamma-release assay (IGRA), chest radiograph, sputum smear, and culture and drug susceptibility testing [12,23,29,54]. A TST measurement of 10 mm or more of induration is considered positive (regardless of prior receipt of the Bacillus Calmette–Guérin (BCG) vaccine) whereas a TST induration of 5 mm or more is considered positive in children with HIV infection, exposure to a patient with active TB contact, or presence of symptoms and signs of TB [23].

In the 2007 U.S. CDC Tuberculosis Technical Instructions (TBTI), chest X-ray is the recommended test for TB screening for all U.S.-bound refugee and immigrants aged 15 years and older; for those with abnormal chest radiographs, symptoms or signs of TB disease, or known HIV infection, further evaluation with three sputum smear plus three cultures for Mycobacterium TB with drug susceptibility testing is recommended [54]. In addition, for all applicants aged 2 to 14 years of age in high-burden countries (WHO-estimated TB incidence rate of ≥20 cases per 100,000 population), screening with TST or IGRA was recommended; for those with positive tests, chest X-ray is recommended [54]. For refugee and immigrant children aged ≤2 years of age, no screening test is recommended (unless the child has symptoms or signs of TB or known active TB adult contact or HIV infection).

Based on 2007 TBTI, children aged 2 to 14 years with a positive TST or IGRA but negative chest X-ray or other evaluation for TB were classified as latent TB infection (LTBI). Children with LTBI can arrive in the U.S. without prior treatment but are recommended to undergo repeat evaluation for TB upon arrival [54]. Evaluation of children with LTBI found high rates of incomplete therapy in conjunction with overdiagnosis based on positive TST tests overseas but negative IGRA after arrival in the U.S. Therefore, IGRA-based TB testing has replaced TST in the 2017 U.S. CDC-based Tuberculosis Technical Instructions (TBTI) for all applicants aged 2 to 14 years of age in high-burden countries (WHO-estimated TB incidence rate of ≥20 cases per 100,000 population) [29,54].

Many refugee children would have received the Bacillus Calmette–Guérin (BCG) vaccine, a common practice in countries with a high incidence of TB. The BCG vaccine has limited efficacy in the prevention of pulmonary TB but is around 80% effective in preventing serious, potentially fatal, disease, such as military TB and TB meningitis in children. Prior receipt of the BCG vaccine is not a contraindication for TST. However, TST may be false positive in children who have previously received the BCG vaccine [55]. In such instances, obtaining an IGRA may be helpful in determining the cause of a “positive” TST (latent TB infection versus BCG vaccine-related) [56]. False-negative TST or IGRA results may be encountered in refugee children who are anergic due to a variety of reasons, such as malnutrition, stress, and untreated HIV infection. Studies have shown that screening for latent TB infection in young migrant children from high incidence countries is also cost effective [57,58,59].

Any child with a positive TST or IGRA warrants further evaluation by performing a physical examination and obtaining a chest radiograph to exclude TB disease. Given the high prevalence of drug-resistant TB in many countries, efforts to isolate the pathogen via obtaining sputum samples (via early morning gastric aspirates or sputum induction) for drug susceptibility testing is crucial [12,23]. Applicants diagnosed with active TB must complete treatment via directly observed therapy prior to arrival in the U.S. [29].

### 2.3. Syphilis

Refugee children and adolescents may be exposed to sexual violence and abuse before resettlement. However, data on the prevalence of syphilis in migrant children is limited [5]. One study from the U.S. has reported a relatively high rate of syphilis seropositivity in refugees arriving from the African region (874 cases out of 233,446 screened corresponding to 373 cases per 100,000); adolescents and young adults from aged 15 to 24 years accounted for 101 (11.6%) of all cases of syphilis seropositivity [60].

Refugee children aged 15 years and older undergo routine screening for syphilis using a two-stage serologic testing procedure at a specified in-country reference laboratory prior to arrival in the U.S. [31]. The initial screening is performed using non-treponemal tests (e.g., Venereal Disease Research Laboratory (VDRL)) or rapid plasma regain (RPR), and if found reactive are confirmed with an appropriate treponemal test. Refugees with reactive test results have to complete treatment for syphilis in accordance with the CDC’s sexually transmitted guidelines prior to arrival in the U.S. [61].

After resettlement, screening for syphilis (and other sexually transmitted infections) is recommended if there is a concern for congenital syphilis, sexual abuse, or positive maternal syphilis serology. Children with reactive syphilis serology should be referred to pediatric infectious disease specialists or health care providers at the local health departments to confirm the diagnosis, disease staging, and treatment; in addition, infection due to other treponemal subspecies (e.g., yaws, bejel, and pinta) must be excluded [23].

### 2.4. Hepatitis A

Hepatitis A virus (HAV) is an endemic disease in many countries of origin of migrant children. Outbreaks of HAV have occurred among refugee children from Syria, Afghanistan, and Iraq living in hosting facilities in Greece, Germany, and other countries [62]. The spread of HAV occurs via the fecal–oral route and predominantly affects school-aged children 5 to 9 years of age [23,63]. Serologic testing of children at the time of the initial visit to the health care provider can determine acute infection [Immunoglobulin (Ig)M antibody to HAV] from immune status (total HAV IgM and IgG). Most children may have natural immunity from prior HAV infection acquired at their country of origin whereas others may be non-immune and candidates for vaccination against HAV. In a study in refugees and asylum seekers in Germany on the immunity against hepatitis A-E viruses, 81% of refugee minors were immune, which corresponds to the high anti-HAV seroprevalence rates in patients from Sub-Saharan and Northern Africa, and from the Middle East [64]. The U.S. CDC and AAP recommends the hepatitis A vaccine as part of the routine immunization schedule for all children aged 1 year and older without evidence of immunity [23].

### 2.5. Hepatitis B

Hepatitis B virus (HBV) infection is a serious public health challenge in many countries, with an estimated 257 million people living with chronic HBV infection worldwide [65]. The Pacific Islands, Southeast and East Asia, Africa, Middle East, and Central and Eastern Europe have the highest incidence of HBV [66]. The transmission of HBV occurs via infected blood or body fluids. The primary routes of transmission of HBV are vertical (mother-to-child) and horizontal early childhood transmission, resulting in the most chronic infection [67]. The reported prevalence of positive HBsAg was 15% in migrants from sub-Saharan Africa resettled in Spain and up to 10% among undocumented migrants in Italy [68,69,70].

Perinatal HBV infection results in chronic liver disease in 90% of children; early mortality is noted in 25% of untreated children with chronic liver disease due to HBV-related cirrhosis and hepatocellular carcinoma [71]. Universal hepatitis B immunization at birth and in infancy is the key strategy for global elimination of HBV infection, and has been highly effective in reducing new vertical infections [72]. In many endemic countries for HBV, infants do not receive the HBV vaccine at birth or early infancy. Therefore, serologic testing to screen for chronic HBV infection using HBsAg is recommended for all immigrant, refugee, and internationally adopted children regardless of immunization status [23]. Studies have shown that migrant serologic screening for HBV infection is also cost effective [73,74].

Local or state health departments must be notified of positive HBsAg results. Children with positive HBsAg test results should be re-tested and additional serologic markers to HBV core Ag performed to differentiate acute (positive IgM anti-HBc) from chronic HBV infection (negative IgM anti-HBc, positive total anti-HBc, and persistence of HBsAg for at least 6 months). [23]. Children diagnosed with chronic HBV infection must be referred to a pediatric infectious disease specialist for further evaluation and management.

### 2.6. Hepatitis C

The burden of hepatitis C virus (HBV) infection is high in China, Russia, and South East Asia, with an estimated prevalence of 0.15% in children 1 to 19 years of age, resulting in 3·5 million people living with HCV infection (95% CI 3.1–3.9 million). [75]. HCV infection is usually asymptomatic in the pediatric age group; in contrast to chronic HBV infection, complications of cirrhosis and hepatocellular carcinoma are unusual.

Serologic testing to screen for HCV is recommended for all immigrant, refugee, and internationally adopted children [23]. Initial screening tests for HCV infection include obtaining a serum enzyme immunoassay (ELISA). However, in children up to 18 months of age, a positive ELISA may reflect passively transferred maternal antibody to HCV. Further evaluation with a recombinant immunoblot assay or polymerase chain reaction for HCV should be considered for confirmation of HCV infection following a positive ELISA test result. Children with HCV infection must be referred to a pediatric infectious disease specialist for further evaluation and long-term follow-up [23].

### 2.7. Vaccine-Preventable Diseases

The high burden of vaccine-preventable diseases among migrant children compared with non-migrant children underscores the need for timely vaccination in this vulnerable population [76,77,78,79,80]. Despite the recommendations for age-appropriate vaccinations for migrant children with absent or uncertain immunization records, studies have shown that migrants in the EU are not up to date on vaccinations [81,82]. A cohort study from Denmark showed that 30% of asylum-seeking children were not up to date on vaccinations in accordance with national guidelines [17]. In another report from Sweden, measles seroimmunity gaps were noted in newly arrived adult immigrants from certain European regions and Russia [83]. An outbreak of measles was reported in 2017 from Minnesota, U.S., which predominantly affected children of Somali descent [84]. Another study among Somali refugees in Minnesota reported that 18% of the study participants were seronegative for varicella, underscoring the need for enhanced education to improve varicella vaccination rates in these at-risk communities [85]. Another study from Minnesota indicated that childhood vaccination coverage at 36 months was 44% in children born to mothers from Somalia compared with 77% in children born to mothers from Central and South America [86]. Declines in MMR vaccine coverage secondary to vaccine safety concerns have been reported in children born to Somali parents in Minnesota compared with children born to non-Somali parents [87]. In conflict settings, outbreaks of polio and other VPDs are a major cause of morbidity and mortality, with the potential to spill over to neighboring nations [88,89,90,91].

### 2.8. Intestinal and Tissue Parasites

Stool examinations for ova and parasites performed at a laboratory by those with expertise in parasitology may yield a parasite in 15% to 35% of migrant children [23]. The prevalence of intestinal parasitic infection varies based on country of origin and age. Commonly diagnosed intestinal parasitic infections are *Giardia intestinalis*, *Cryptosporidium* species, *Ascaris lumbricoides*, and *Trichuris Trichura* followed by *Strongyloides stercoralis*, *Entamoeba histolytica*, and hookworm [92]. Newly arrived migrants with complaints of diarrhea should be screened for infection due to *Cryptosporidium* species and other invasive bacterial pathogens, including *Salmonella*, *Shigella*, *Camplylobacter*, and diarrheagenic *E. coli* species (including Shiga toxin-producing *E. coli*).

Since 1999, the Centers for Disease Control and Prevention (CDC) recommended a single dose of presumptive albendazole treatment for intestinal parasites, administered overseas for U.S.-bound refugees. This approach has resulted in a significant decline in the prevalence of intestinal helminths (from 22.5% to 7.5%) among newly arrived refugees from Africa and Southeast Asia [93]. The CDC has published treatment guidelines for intestinal parasites based on prior receipt of presumptive albendazole therapy in U.S.-bound refugee children [34]. A total of three stool specimens collected on different days for ova and parasites is recommended for the screening of intestinal parasitic infection among all immigrant, refugee, and internationally adopted children arriving in the U.S. regardless of nutritional status or presence of symptoms. [23]. However, data from Germany indicate no evidence of the benefit of routine stool screening to detect intestinal parasites among refugee minors [94]. Tissue-invasive parasitic infections, such as schistosomiasis and strongyloidiasis, must be included in the differential diagnosis of recently arrived immigrants and refugee children with unexplained eosinophilia (defined as an absolute eosinophil count greater than 450 cells/µL with negative stool testing for ova and parasites) [23,28,95,96]. Around 30 to 250 million people have schistosomiasis and strongyloidosis in endemic countries [4].

A high prevalence of schistosomiasis (9% to 60%) has been reported among adolescent migrants from sub-Saharan Africa re-settled in Germany, Switzerland, Spain, and Canada [53,95,97,98]. *Schistosoma mansoni* and S. *haematobium* are the two primary species of Schistomas causing intestinal and genitourinary disease, respectively. Serious long-term complications of untreated schistosoma infection include hepatic cirrhosis, portal hypertension, bladder and ureter fibrosis, hydronephrosis, and bladder cancer [99,100]. Serologic testing for schistosoma is very sensitive and is recommended for all migrant children from endemic countries who have evidence of eosinophilia, negative stool ova and parasite examination, and exclusion of common infections associated with eosinophilia [23,31,33]. However, an eosinophil count may not be a good screening parameter for schistosomiasis and universal serological screening might be more beneficial; in one study, 25% of refugees from sub-Saharan Africa had schistosomiasis but only 7.7% had a high eosinophil count [98]. The positive- and negative-predictive value of the eosinophil count is poor and therefore, some experts suggest schistosomiasis serology in all migrant children from high-endemic countries, regardless of their eosinophil count [23]. In some settings in the EU, circulating-cathodic-antigen (CCA) rapid point-of-care assay are available [101]. In refugee children aged 2 years and older with eosinophilia, serologic testing for lymphatic filariasis is a consideration if they have arrived from countries endemic for lymphatic filariasis [23,31,33].

*Stronglyloides stercoralis* is an intestinal parasitic disease, prevalent in many parts of the world, with a propensity to cause life-threatening disease, especially in immunocompromised hosts after a prolonged period of subclinical infection [102]. Serologic testing for strongyloides is recommended for all refugee children with unexplained eosinophilia regardless of country of origin [23,31,33,95].

### 2.9. Chagas Disease (American Trypanosomiasis)

Chagas disease, also known as American trypanosomiasis, is a zoonotic tropical infection caused by a protozoan parasite *Trypanosoma cruzi*. Most infections in humans occur via vector-borne transmission through infected triatomine insects in endemic locations; other routes of transmission include vertical from mother to baby or receipt of blood transfusion in a country with endemic Chagas disease. The disease is endemic in Mexico, and Central and South America and estimated to affect 6 to 8 million people in the Americas, including 326,000 to 347,000 in the U.S. [103]. Following a prolonged asymptomatic phase, chronic CD can result in serious cardiac and gastrointestinal complications in approximately 30% to 40% of infected patients. Cases of CD have been reported from migrants arriving from Latin America to the U.S. and Canada, but the disease seems to be rare in Europe, although the diagnosis may be missed due to a lack of knowledge and awareness among patients and providers in non-endemic countries.

In suspected cases, the diagnosis is confirmed by serology by detection of IgG antibodies against *T. cruzi* utilizing at least two different assays (such as ELISA, indirect immunofluorescent, or indirect hemagglutination). Serologic screening for Chagas disease is only recommended for children older than 12 months due to the potential interference by the persistence of maternal antibody [23].

### 2.10. Malaria

Sub-Saharan Africa has the highest burden of malaria, with more than 90% of malaria cases and deaths, primarily in children aged less than 5 years [104]. The benefit of routine post-arrival screening for malaria in asymptomatic cases is unclear and not recommended given the limited sensitivity of diagnostic tests, such as blood films and rapid antigen tests [5,12].

U.S.-bound refugee children from sub-Saharan Africa that are endemic for *Plasmodium falciparum* malaria would have received pre-departure presumptive treatment with artesunate combination therapy unless contraindicated in certain specific groups (e.g., pregnant or lactating women, children with body weight less than 5 kg at time of departure). U.S.-bound refugee children from a malaria-endemic country or from sub-Saharan Africa who present with a febrile illness should be promptly evaluated to exclude malaria [105].

### 2.11. Other Infections

Other infectious diseases, especially of the skin, such as impetigo, candidiasis, tinea, scabies, and pediculosis are frequently diagnosed in refugee and internationally adopted children and adolescents, reflecting unhygienic living situations, overcrowding, and social marginalization [23,106]. Outbreaks of vaccine-preventable diseases (e.g., measles), and gastrointestinal and cutaneous infections have been reported in the early settlement period [107,108]. Health care workers must be aware of the clinical presentations of other tropical infectious diseases prevalent in the refugee country of origin, such as typhoid fever, Zika, cysticercosis, echinococcosis, leprosy, cutaneous diphtheria, chronic helminthiasis, and louse-borne relapsing fever [24,109].

## 3. Prevention

### Vaccination Strategies

As part of the medical examination, all U.S.-bound refugees undergo an assessment of vaccine-preventable diseases (polio, tetanus, diphtheria toxoids, pertussis, *Haemophilus influenza* type b, rotavirus, mumps, measles, rubella, hepatitis A, hepatitis B, meningococcal disease, influenza, pneumococcus, and varicella). However, refugees and internationally adopted children can enter the U.S. with an incomplete immunization schedule. Based on the U.S. Immigration and Nationality Act, there is no requirement for U.S.-bound refugees to meet immunization requirements. However, proof of immunization status is required for refugees already residing in the U.S. and applying for an adjustment of status for permanent residency, usually 1 year after arrival.

Clinicians should review immunization records, if available, to determine if the vaccine doses and intervals are consistent with the age-appropriate immunization recommendations of the U.S. Advisory Committee on Immunization Practices (ACIP) and other national guidelines [23,31,110,111,112].

An accurate assessment of immunization status in refugee children is challenging because of an unreliable history and uncertainty in the clinical diagnosis of vaccine preventable diseases (VPDs), such as measles, mumps, rubella, and varicella. Assessment of immunity against VPDs by measurement of antibody titers in refugees is limited. One study found that sero-testing for varicella immunity was more cost effective compared with universal administration of the varicella vaccine in refugee children [113]. Vaccination without obtaining serologic tests for VPDs may be a consideration in some settings since tests may be expensive, have a delayed turnaround time, and patients may be lost to follow-up due to re-location.

According to the U.S. Immigration and Nationality Act of 1996, for refugees with absent records or incomplete immunization status, a single vaccine dose in a series recommended by the U.S. Advisory Committee on Immunization Practices (ACIP) suffices for the immigration process, with a plan to complete the remaining doses in a series and catch-up vaccinations [23,31,113].

## 4. Knowledge Gaps, Research Agenda, and Future Directions

Public health programs are crucial for implementation of the screening and evaluation of migrants for infectious diseases. Historically, screening and quarantine procedures were performed utilizing port-of-entry strategies at the time of arrival of ships [114]. However, given the different routes of travel and dramatic increase of newly arriving migrants, the effectiveness of this strategy is limited [115]. For more than two decades, the U.S. Centers for Disease Control and Prevention’s Division of Global Migration and Quarantine has implemented a health assessment framework (overseas screening, treatment, and immunization programs) to improve health for US-bound immigrants and refugees [29]. This program has had many successes, including decreased TB rates in the U.S., decreased transmission and importation of VPDs, reduction in morbidity from parasitic diseases, and reduced domestic healthcare costs [116].

Migrant health reviews from Canada indicate that disease risk is affected by many factors, such as gender, forced migration, and migrant country of origin, and provide important guidance to develop evidence-based evaluation and vaccination strategies [117]. Similar migrant health reviews from Ireland, Australia, and other countries have also provided evidence to inform public health policy and primary care assessments [114,115,117,118]. Data from the Migration Integration Policy Index health system survey indicate that evidence-based programs, guidelines, and policy for infectious disease, mental health, and maternal health, and chronic disease evaluation of migrants is limited and warrants further studies in many countries in Europe [4,119,120]. The cost effectiveness of the implementation of screening and treatment of latent TB infection and effectiveness to prevent active TB disease is an area of future research [121]. The development of evidence-based guidance for screening, treatment, and prevention of infectious diseases, including vaccine-preventable diseases, in newly arrived migrants is a major public health priority of the European Centre for Disease Prevention and Control (ECDC) [4,5].

The United Nations Convention on the Rights of the Child state that migrant children must receive the same standard of health care as received by the local population [122]. A recent review on migration and infectious diseases highlights the importance of screening programs tailored to various steps in the migratory pathway and improve access to care irrespective of the legal status of the individual [22]. However, engaging refugee and other migrant populations in health care and prevention services remains a challenge due to many barriers to accessing health care services, such as high rates of non-insurance, lack of health information, language and cultural differences, transportation issues, stigma, discrimination, and social isolation [10,123,124].

The reported outbreak of measles among Somali children in Minnesota who were not up to date on measles-mumps-rubella (MMR) vaccinations due to safety concerns (erroneous link to autism) highlights the need to develop enhanced community outreach and education with families and Somali community leaders to address vaccine hesitancy, provide health education, and improve vaccination rates [86]. Studies with a rigorous study design must be conducted to assess interventions to successfully implement vaccination programs for migrant populations [125,126]. In conflict zones and complex humanitarian emergency settings, the implementation of mass immunization campaigns has resulted in controlling the outbreaks of wild polio virus and circulating vaccine-derived poliovirus infections, suggesting that innovative approaches to vaccinate children on the move are needed [127,128]. Cost-effective interventions to address health care disparities and provide high-quality primary and secondary health care for large numbers of recently arrived migrants remains a major priority for many high-income countries [10,11,28,129,130,131,132].

An estimated 18 million children reside in the U.S. with at least one immigrant parent; 4.5 million children are U.S. citizens with mixed immigration status with at least one family member with undocumented status [133]. Recent changes in U.S. immigration policy related to unaccompanied children, family separation, and detention of people arriving from El Salvador, Honduras, and Guatemala at the Southern border of Mexico has been a major concern [134]. Strong advocacy efforts and policy statements by the American Academy of Pediatrics provide recommendations for the care of immigrant children following release from detention facilities to address medical and legal needs, education, and interpretation services. [135,136]. Increased funding in conjunction with multi-sector collaboration between governments, nongovernmental organizations, and local community agencies are needed to address the complex social, health, and economic needs of refugee and immigrant children and youth [137,138].

## 5. Conclusions

Infectious diseases often threaten the health of migrant populations and host communities. Key priority infectious diseases among refugee populations include tuberculosis, hepatitis B, and vaccine-preventable and parasitic diseases. Although data on the health risks and needs of refugee exists in some high-income countries, there is an urgent need to develop robust evidence-informed guidance on screening for infectious diseases and vaccination strategies on a broader scale to inform national policies. Innovative approaches to reach migrant communities in the host nations, address health and other complex barriers to improve access to high-quality integrated health services, and strong advocacy to mobilize resources to improve health, safety, and wellbeing of refugee children and their families are urgent priorities.

## Figures and Tables

**Table 1:** Top 10 Countries of origin with the highest refugee arrivals to the United States in 2017 and 2018.

2018		2017	
Country of Birth	No (%)	Country of Birth	No (%)
Dem. Rep. Congo	7878 (35.0)	Dem. Rep. Congo	9377 (17.5)
Burma	3555 (15.8)	Iraq	6886 (12.8)
Ukraine	2635 (11.7)	Syria	6557 (12.2)
Bhutan	2228 (9.9)	Somalia	6130 (11.4)
Eritrea	1269 (5.6)	Burma	5078 (9.5)
Afghanistan	805 (3.6)	Ukraine	4264 (7.9)
El Salvador	725 (3.2)	Bhutan	3550 (6.6)
Pakistan	441 (2.0)	Iran	2577 (4.8)
Russia	437 (1.9)	Eritrea	1917 (3.6)
Ethiopia	376 (1.7)	Afghanistan	1311 (2.4)

Data from [13].

**Table 2:** Key priority health issues in refugee children.

Health Issue	
Growth and Development Issues	Various forms of Protein-Energy Malnutrition
Learning Difficulties	Limited data available on educational outcomes
Vision and Hearing Impairments	Refractory errors, presbyopia
Oral Health	Dental caries
Vaccinations	Risk for measles and other vaccine-preventable diseases
Nutritional Disorders	Iron deficiency anemia Micronutrient deficiency Malnutrition Vitamin D deficiency
Mental Health	Depression Anxiety Posttraumatic stress disorder
Toxin Exposure	Lead Environmental pollution Prenatal exposure to alcohol
Infectious Diseases	Tuberculosis Hepatitis A, B, C Syphilis Vaccine preventable diseases Intestinal and tissue parasites *Giardia intestinalis*, *Cryptosporidium* species, *Ascaris lumbricoides, Trichuris trichura*, *Entamoeba histolytica*, hookworm, Schistosomiasis and Strongyloidiasis Chagas Disease (American Trypanosomiasis)

**Table 3:** Screening tests for infectious diseases in refugee children, international adoptees, and immigrant children in the U.S. [23].

Disease	Screening Test	Comments
HIV	Serology (HIV 1 and 2)	Virologic tests (HIV DNA or RNA PCR) for children up to 18 months of age
Hepatitis A	Serology	
Hepatitis B	Serology	
Hepatitis C	Serology	
Syphilis	Serology	Nontreponemal tests (e.g., RPR, VDRL, or ART) Treponemal tests (e.g., MHA-TP, FTA-ABS, CIA, or TPPA)
Tuberculosis	TST or IGRA £ Chest X-ray *	Medical History and Physical Examination Sputum smears and cultures for individuals with an abnormal chest radiograph Drug susceptibility testing for individuals with positive TB cultures Completion of directly observed treatment prior to immigrant for individuals with pulmonary disease
Intestinal Parasites	Stool examination	Three specimens on separate days with requests for *Cryptosporidium* and *Giardia* species testing
Tissue Parasites:		
*Schistosomiasis* spp., *Strongyloides* spp., *Toxocara canis* Lymphatic filariasis €	Serologic testing	Screening for tissue parasites suggested in children with eosinophilia (absolute eosinophil count >450 cells/mm^3^) and negative stool ova and parasite examination

Abbreviations: HIV, human immunodeficiency virus; IGRA, interferon-gamma release assay; PCR, polymerase chain reaction; TST, tuberculin skin test; £ IGRA-based TB testing has replaced TST in the 2017 Tuberculosis Technical Instructions (TBTI) for all applicants aged 2–14 years of age in high burden countries (WHO-estimated TB incidence rate of ≥20 cases per 100,000 population). * Chest X-ray is recommended for TB screening for all applicants aged 15 years and older. € Screen for lymphatic filariasis in children aged >2 years from endemic countries.

**Table 4:** Professional society and organizations with key resources for clinicians caring for immigrant, refugee, and internationally adopted children.

American Academy of Pediatrics (AAP)	AAP Immigrant Child Health Toolkit
Canadian Collaboration for Immigrant and Refugee Health (CCIRH)	Refugee health e-Learning, Evidence-based guidelines
lefts for Disease Control (CDC)	Medical examination of refugees and immigrants, Refugee health guidelines and profiles, Laws and Regulations
International Organization for Migration (IOM)	World Migration Report, Migration Profiles, Situation Reports
Minnesota Department of Health (MDH)	left of Excellence in Refugee Health, Online interactive tool for CDC’s domestic health screening guidance for individual refugees
Refugee Health Technical Assistance left (RHTAC)	Best Practices for Communication to Refugees through Interpreter, Refugee suicide prevention training toolkit, Community Dialogue
United Nations Children’s Fund (UNICEF)	Research and reports, Stories from the field,
United Nations High Commissioner for Refugees (UNCHR)	Global Report, Asylum Resources, Protection Manual
World Health Organization (WHO)	Global Action Plan for promoting the health of refugees and migrants, Migrant Clinicians Network
